# Relevance of immunohistochemical expression of p57^kip2^ in epithelial ovarian carcinoma- A systematic literature review

**DOI:** 10.1186/1757-2215-5-46

**Published:** 2012-12-21

**Authors:** Thumuluru Kavitha Madhuri, Anil Tailor, Ben Haagsma, Helen Coley, Simon Butler-Manuel

**Affiliations:** 1 Department of Gynaecological Oncology, Royal Surrey County Hospital NHS Foundation Trust, Guildford, Surrey GU2 7XX, UK; 2 Faculty of Health & Medical Sciences, University of Surrey, Guildford, UK; 3 Department of Histopathology, Royal Surrey County Hospital NHS Foundation Trust, Guildford, Surrey, GU2 7XX, UK

**Keywords:** Epithelial ovarian cancer, Pathobiology, Histology, Immunohistchemistry, Cell cycle, Cyclin dependant kinase, p57^kip2^

## Abstract

**Background:**

Epithelial Ovarian Cancer (EOC) is the second most common gynaecological cancer and accounts for more deaths than all gynaecological cancers combined. Despite extensive research, progress has been slow in understanding the pathobiology. EOC is identified as a heterogeneous malignancy with various histological subtypes. It is now well known that these different histological subtypes show differences in terms of presentation, response to treatment, immunohistochemical (IHC) reactivity and molecular profiling. Cell cycle deregulation is key in cancer development and there is some evidence in the literature that this is relevant to the problem of EOC and the development of drug resistant disease. The need to identify prognostic markers has led to several gene profiling studies using tumour tissue with equivocal results. p57^kip2^ is one such cell cycle regulator and its functions are being explored as recent research has shown that it is more than just a negative regulator of the cell cycle.

**Aims:**

The aim of this review is to evaluate the literature around the IHC expression of p57^kip2^ in EOC.

**Methods:**

Systematic review of the literature focussing on clinical outcome and immunohistochemical expression in epithelial ovarian cancer.

**Results:**

Four papers are discussed in this review and have shown great variation in IHC expression of p57^kip2^ in EOC. These studies incorporated different histological subtypes of EOC. However they all suggest that p57^kip2^ has a significant role in prognosis and its therapeutic indication needs to be studied. Multicentre collaborative studies on individual histological subtypes might provide more data and help to increase the number of cases especially for rarer tumours.

## Introduction

In 2008, epithelial ovarian cancer (EOC) accounted for 225,000 new cases worldwide and causes 140,100 deaths [1]. EOC is the second most common gynaecological cancer and accounts for more deaths than all gynaecological cancers combined. In the UK, there were 6,537 new cases of ovarian cancer diagnosed in 2008 and 4,440 women died of ovarian cancer in 2008 [2]. Treatment for EOC includes surgery and chemotherapy. Although there have been some advances in treatment and management of this disease, more than 75% of cases at presentation are advanced Stage 3/Stage 4 with a very poor 5 year survival (20-30%) [3].

Despite extensive research, progress has been slow in understanding the pathobiology of the cause and progression of EOC. In their landmark paper on the hallmarks of cancer, Hanahan and Weinberg suggested six essential cell physiology alterations that led to cancer development and followed this with an updated review considering 2 further enabling characteristics including genetic instability and tumour promoting inflammation together with avoiding immune destruction [4,5]. In particular, and in relation to the present review, the evasion of growth suppressors and the concomitant drive on proliferation involving disruption of the activities of RB and/or p53 have been the subject of many research studies. Hanahan and Weinberg included a focus on therapeutic targeting of the hallmarks of cancer involving cyclin-dependent kinase inhibitors, which is particularly relevant to this review [5].

Cell cycle deregulation is key in cancer development and there is some evidence in the literature that this is relevant to the problem of EOC and the development of drug resistant disease [6]. Despite good initial response to platinum-based chemotherapeutic agents, prognosis with EOC is poor as there are few effective chemotherapeutic agents available for management of this disease. Moreover, there is a need to identify patients who are likely to relapse whilst undergoing chemotherapy treatment with paclitaxel and carboplatin. The need to identify prognostic markers has led to several gene profiling studies using tumour tissue with equivocal results. One of the criticisms levelled at such studies has been the possibility for contamination of tumour tissue with stromal components. However, it would seem that less effort appears to have gone into identification of molecular markers of prognosis using immunohistochemistry (IHC) of tissue sections and correlation with clinical outcome. This approach has the advantage in that the tumour component of the tissue can be clearly identified.

EOC is identified as a heterogeneous malignancy with various histological subtypes. It is now well known that these different histological subtypes show differences in terms of presentation, response to treatment, IHC reactivity and molecular profiling. Molecular studies have demonstrated this genetic heterogeneity. However, problems have been posed in previous studies that grouped together all subtypes when evaluating the expression of the various cell cycle regulators [7]. Recognition of this has now led researchers down the path of exploring the individual cell cycle regulators in various subtypes of cancers. p57^kip2^ is one such cell cycle regulator and its functions are being explored as recent research has shown that it is more than just a negative regulator of the cell cycle. The aim of this review is to evaluate the literature around the IHC expression of p57^kip2^ in EOC. Firstly, p57^kip2^ and its role in tumorigenesis will be discussed with emphasis on EOC followed by evaluating the reported IHC expression of p57^kip2^ in EOC to understand the role of p57^kip2^ better, and finally correlating the IHC expression with reported clinical outcomes.

### Cell cycle and p57^kip2^

**Figure 1:**
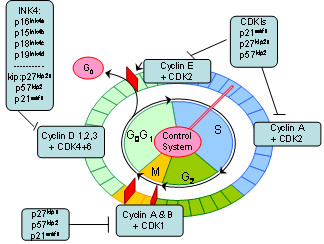
Cell Cycle with various cell cycle regulatory proteins.

The cell cycle is divided into 4 phases: G1, S, G2 and M. Various cell cycle regulatory proteins act at different phases in the cycle as shown in Figure 1.

Changes in cell cycle regulation can lead to uncontrolled cell proliferation and oncogenesis. The progression of the cell cycle through the various phases is tightly regulated by cyclins and cyclin-dependent kinases (CDKs) and simultaneously counterbalanced by cyclin dependent kinase inhibitors (CDKIs). The transition from the G1 to the S phase is regulated by the CDKIs. The CDKIs are further subdivided into the INK4 family consisting of p16^ink4a^, p15^ink4b^, p18^ink4c^ and p19^ink4d^ and the CIP/KIP family consisting of p21 ^waf1^, p27 ^kip1^ and p57^kip2^. The CIP/KIP family regulates the cell cycle both positively and negatively by interacting with Cyclins A, D & E. Hence p57^kip2^ levels are highest in the G0 & G1 cell cycle phases in keeping with its role as a CDKI. Its levels also steadily decrease while progressing from G1 through the S cell cycle phase and over expression of p57^kip2^ induces G1 arrest in cultured cells.

In humans, the *p57*^*kip2*^ encoding gene (correctly called the *CDKN1C* gene) is located on chromosome 11p15.5 and is maternally expressed and paternally imprinted [8]. p57^kip2^ is distributed in the nucleus, however, cytoplasmic distribution has been reported in cancer tissue [8]. It is an important negative regulator and was previously considered to be a tumour suppressor. Recent findings of its involvement throughout the regulation of cell cycle processes suggest a multifaceted existence of the p57^kip2^ protein [9].

### p57^kip2^ and embryology

p57^kip2^ has been found to be essential for normal development. It is found in derivatives from all the three germ cell layers and appears to be irreplaceable by other CDKIs. This has been demonstrated in mouse models wherein *p57*^*kip2*^ devoid mice die within 2 weeks of age showing dysregulation in cell proliferation, differentiation and apoptosis as compared to other CIP/KIP family proteins which do not appear to be lethal suggesting that the p57^kip2^cellular function far exceeds that of its CDKI counterparts [8]. Rodent data suggests that p57^kip2^ is highly cell-specific with a temporal and spatial p57^kip2^ expression during embryonic and fetal development and continuing on into puberty and adulthood. The highest and maximum widespread tissue distribution occurs during organogenesis when p57^kip2^ is present in all major organs with its expression peaking at key differentiation stage for each organ. Levels subsequently appear to be undetectable in several organs after birth. Adult human tissue expressing significant p57^kip2^ mRNA levels include skeletal muscle, brain, heart, lung, kidney, pancreas, testis and placenta with lower expression noted in stomach and ovaries [10].

### Altered p57^kip2^ and cancer

Abnormal p57^kip2^ function includes its participation in cancer initiation and progression. Since p57^kip2^ was discovered as a CDKI, research revolved around describing its ability to inhibit proliferation hence rendering it a tumour suppressor gene status [6]. p57^kip2^ has also been shown to influence cell differentiation thus positively inhibiting cancer progression and preventing their maintenance in an undifferentiated state. Evading cell death is a significant ability of cancer cells. Conflicting reports have been put forth relating to the apoptotic role of p57^kip2^. Both pro and anti-apoptotic functions have been described depending on cellular context, regulatory tumour cell pathways and tumour microenvironment. However, this evidence is from mouse models with structural differences compared to humans. Clinical studies suggest that absence of p57^kip2^ abrogates the pro-apoptotic function of anti-cancer therapy resulting in poor prognosis due to drug resistance development. Vlachos et al. demonstrated sensitization of cancer cells to apoptotic agents such as cisplatin, etoposide and other agents by selective p57^kip2^ expression [11].

Cancer metastasis is the ability of the cells to travel and invade neighbouring tissues. This involves regulation of cytoskeletal dynamics, particularly through the Rho family and a negative feedback loop has been identified [12]. Nan et al. showed that decreased p57^kip2^ expression was associated with increased invasive and metastatic tumour potential in hepatocelluar carcinoma [13]. Angiogenesis is another adaptive process of solid tumours to supply themselves with nutrients and oxygen for their further growth. Matsuura et al. demonstrated increased VEGF (vascular endothelial growth factor) expression in placentae of p57^kip2^ null embryo mice suggesting a reciprocal correlation between lack of p57^kip2^ and VEGF expression though further evidence is awaited [14].

Epigenetic control is important in p57^kip2^ regulation frequently via promoter methylation of CpG islands [6]. Transcriptional and translational down-regulation of p57^kip2^ has been demonstrated by Pateras et al. in various cancers with decreased expression associated with aggressiveness [10]. Histone modifications of p57^kip2^ repression helps participate in tumorigenesis in methylation dependent and in an independent manner. Several studies have shown that p57^kip2^ gene reactivation after demethylating agent treatment in many cancers demonstrates the epigenetic mechanism of downregulation [6,10].

### p57^kip2^ and pathology

p57^kip2^ has been extensively studied in Beckwith-Wiedemann Syndrome (BWS) which is a heterogenous disease. Clinical manifestations of pre-eclampsia have been displayed by mutant mice defective for the maternal p57^kip2^ allele. Absence of p57^kip2^ in complete hydatidiform moles which is a trophoblastic proliferation abnormality suggests that p57^kip2^ is a very sensitive marker to differentiate between complete and partial hydatidiform mole since p57^kip2^ is a paternally imprinted and maternally expressed gene, it is found in partial moles but absent in complete molar pregnancies [15].

The role of p57^kip2^ has been studied to a small extent in cardiovascular and renal pathology as well. Several IHC studies have been carried out evaluating p57^kip2^ expression in lung, oral, gastrointestinal tract (oesophageal, gastric, colorectal), hepatocellular and pancreatic malignancies [10]. Despite studies highlighting the importance of p57^kip2^ in the female genital tract pathology, few studies have highlighted the role of p57^kip2^ as a prognostic molecular marker for EOC as compared to studies reported on p27^kip1^ and p16 [16].

Data currently available are limited and conflicting and the role of p57^kip2^ as an IHC marker of prognosis requires clarification.

Based on the above considerations, we conducted a systematic review with following meta-analysis to clarify the association. In this meta-analysis, we examine studies with IHC data following p57^kip2^ use. Furthermore, any correlation with clinical outcome is also evaluated to assess the efficacy and usefulness of p57^kip2^ as a potential prognostic marker.

## Methods

### Search strategy

Thorough literature search of MEDLINE, EMBASE, and COCHRANE electronic databases was performed by 2 independent reviewers to identify relevant studies. This included bibliographies to identify all additional studies. Hand searching of the grey literature was also carried out to identify any relevant conference abstracts and unpublished MD/PhD theses. The search was not limited to any time duration. To enable assimilation of all relevant published research, all search terms were expanded and all sub-categories were included. The exact syntax of search terms included ovarian carcinomas as well as cell membrane protein and other mesh terms.

Initial scanning of the title and abstract was performed to exclude any unrelated studies. Reading of the full text of all remaining articles was performed to determine the extent of information contained in them on the subject of interest.

### Selection criteria

Inclusion into the current review was based on the following criteria for all retrieved studies:

Tumour site of study performed was ovary. Papers were reviewed to determine whether IHC assessment of p57^kip2^ had been performed. The studies reported the results of the IHC scoring and an attempt had been made to correlate this with clinical outcomes. Due to the small number of studies, reviews that explained the role of p57^kip2^ in EOC have also been included following agreement between the authors.

Studies that did not involve EOC, performed only on cell lines, western blotting and gene profiling studies (non IHC studies) have not been reviewed.

### Identified studies

**Figure 2:**
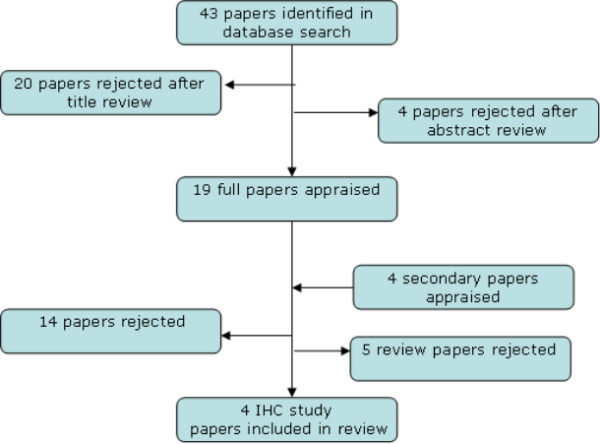
Filtering of studies and final number of studies included in the review.

Search of the databases using the above search terms led to identification of a total of 43 papers. All the 43 papers were independently assessed, 20 of which were excluded by title alone as not being relevant. 4 papers were rejected following review of the abstracts as p57^kip2^ was discussed in relation to molar pregnancy in 3 of these [16-18]. The 4^th^ paper discussed p57^kip2^ in relation with apoptosis and cancer [19]. Extraction onto a datasheet led to further elimination due to duplication of 2 studies. Full manuscripts of all the remaining studies were reviewed. 4 secondary papers were appraised from references of the full studies. 14 papers were rejected and a further 5 review papers have also been rejected for the purposes of this review. Consequently 4 full papers are included in the final review as only 4 IHC studies were identified [20-23]. Results of the search and subsequent filtering of the studies has been shown in Figure 2.

### Data extraction

Information from the studies was extracted by 2 researchers (TKM & BH) using the data extraction form. Both researchers were in agreement regarding the studies and the data collected included first author’s name, country study was performed in, year of publication, no of cases, IHC findings and clinical outcome if recorded.

## Results

The four included studies are tabulated as shown in Table 1.

**Table 1:** Showing the 4 studies with IHC performed

**Author**	**Year**	**Country**	**IHC performed**	**No of EOC cases**	**Correlation with clinical outcome**
Rosenberg E	2001	USA	Yes	53	yes
Sui L	2002	Japan	Yes	47	yes
Khouja MH	2007	Norway	Yes	171	yes
Guo J	2011	China	Yes	103	No

The first ever study to evaluate the role of p57^kip2^ using an IHC approach in EOC was performed by Rosenberg et al. in 2001 [20]. For comparison purposes, the cases were divided into 2 groups comparing long term (>5yrs) vs. short term (<2yrs) survivors. All cases included had a confirmed diagnosis of ovarian adenocarcinomas. Women with FIGO stage 1 and stage 4 were excluded from this study. Also excluded were women over the age of 80 at primary diagnosis and cases where an ECOG performance status of 3 or greater had been recorded. IHC on 53 cases of treatment naïve (chemotherapy/radiotherapy) women following surgery was performed. They included 23 short term and 30 long term survivors. The histological subtypes of the included cases are recorded in Table 2.

**Table 2:** Showing histological subtypes of the 4 studies

**Author**	**No of EOC cases**	**Histological subtype**	**Scoring**
Rosenberg E	53	Papillary serous 34	Low vs high Expressors
		Poorly differentiated 10	
		Endometrioid 1	
		Clear cell 3	
		Mucinous 5	
Sui L	47	Not mentioned	Low vs high Expressors
		21 Grade1	
		13 Grade 2	
		13 Grade 3 incl 2 undifferentiated	
Khouja MH	171	Serous 127	4 levels of scoring
		Endometrioid 14	
		Clear cell 8	
		Mucinous 5	
		Undifferentiated 2	
		Mixed NOS 1	
		Mixed without clear cell 4	
		Mixed with clear cell 4	
		Unclassifiable adenoca 6	
Guo J	55	Serous 25	Positive vs negative
		Mucinous 7	
		Endometrioid 11	
		Clear cell 1	
		Undifferentiated 1	
		Non epithelial 8	
		Immature teratoma 1	
		Granulosa Cell 3	
		Dysgerminoma 2	
		Blastoma 1	
		Metastatic cancer 1	
		(site not defined)	

Immunohistochemical stain assessment was positive for distinct brown staining and divided into 5 categories; negative or less than 5% positive, 5% -25% positive, 26%-50%, 51-75% and greater than 75%. These were then categorised as low and high immunopositive tumour cell expressors if less than or greater than 50% respectively. This study suggested that there was no association between p57^kip2^ expression and survival status as compared to the other cell cycle regulators assessed in this study (Cyclin D1, Cyclin E) Table 3.

**Table 3:** Patient Demographics recorded in the studies is tabulated below

**Author**	**No of EOC cases**	**Age yrs**	**Treatment status at surgery**	**FIGO Stage of EOC**	**Grade (G)**	**Other mentioned criteria**
Rosenberg E	53	<80	Naïve	2 & 3 only	only G3 mentioned	ECOG status
Sui L	47	16-77 Median 49	Naïve	1-4	G1 vs G2/3	Nodal status, ascites, residual disease
Khouja MH	171	21-70 Median 54	Naïve	3 only	G1 vs G2/3	Ascites, residual disease and response to chemotheraphy
Guo J	103	18-66 Median 42	Naive	1-4	G1/G2/G3	Nodal status

The following year, Sui et al. published their results on p57^kip2^ expression and its associated clinical relevance in EOC. 103 cases of ovarian tumours were assessed which included 33 benign tumours, 23 borderline and 47 malignancies [21]. Of the 47cases, 16 women were diagnosed with Stage 1 disease, 3 Stage 2, 16 and 12 in Stage 3 and 4 respectively. Grading of the 47 tumours revealed 21 G1, 13 each in the G2 and G3 category. All 47 were treatment naïve. The ratio of positive nuclear staining to the total number of counted cells was recorded and immunopositivity was classified as low or high as in the Rosenberg study with 50% being the cut off. The investigators at this stage were blinded to the clinical outcome. Highest positivity was recorded in benign tumours and lowest in the malignancies (Table 4). A statistically significant correlation was found between p57^kip2^ expression and stage and grade of tumour. Median follow-up in this study was 24 months (range 2–156) and low p57^kip2^ expression was found to be associated with poor survival suggesting a linkage to a more aggressive phenotype of EOC in this category. This study concluded p57^kip2^ IHC may confer important prognostic implications for EOC Table 4.

**Table 4:** Showing results of the 4 studies

**Author**	**p57**^**kip2**^**Positivity in EOC**	**Benign tumours**	**Borderline tumours**	**Clinical outcome**
Rosenberg E	48/53 n/a	n/a	n/a	No relation to survival status
Sui L	19/47	21/33 Normal ovarian tissue expression was positive (data not included)	12/23	Significant correlation between p57kip2 expression and tumour grades/clinical stages noted. Low p57kip2 expression associated with poor survival. Important prognostic implications exist
Khouja MH	<10%Positivity in 130/171 & >10% in 41/171	n/a	n/a	No correlation exists with clinical and prognostic outcomes
		0/10 positivity in normal Ovarian tissue		
Guo J	15/55	12/15 Normal ovarian tissue expression was positive in 16/17	6/8	Not described as clinical correlation not assessed

In 2007, Khouja et al. published their experience with p57^kip2^ and other proteins in 171 treatment naïve patients with Stage 3 EOC (Stage 3a- 13, 3b −20 & Stage 3c −138) [22]. Serous adenocarcinoma was the predominant subtype (127/171) and the other subtypes are shown in Table 2. 156/171 consisted of grade 2/3 tumours and the remaining cases (15/171) were Grade 1. Distinct nuclear staining was considered positive. Semiquantitative scoring classes were adopted depending on the number of positive stained tumour cells with 4 scoring groups; no staining, less than 10% positive, between 10% and 50% positive and more than 50%. The IHC results for p57^kip2^ was negative in 19% of the cases and more than 50% staining was obtained in only 2% (4 cases). This study concluded that there was no correlation between p57^kip2^ and clinical and prognostic outcomes, Table 4.

The final study by Guo et al. in 2011 evaluated 103 ovarian tissues [23]. Of these, 17 were normal ovaries, 15 benign, 8 borderline, 55 EOC and 8 non-epithelial tumours as shown in Table 2. Positive staining was defined as brown-yellow granules distributed in either the nucleus or cytoplasm with higher intensity of stain than the nonspecific background. Greater than 20% scoring over 5 fields was a positive result.

This study principally focussed on EZH2 (enhancer of zeste homolog 2) and compared p57^kip2^ IHC expression since p57^kip2^ is a direct target of EZH2 as shown in other studies. No assessment of correlation p57^kip2^ and clinical outcome was looked into. The study concluded that EZH2 depletion increased p57^kip2^ expression indicating that EZH2 acts as an EOC oncogene by targeting p57^kip2^ Table 4.

## Discussion

The above 4 studies demonstrated varying results. There are several factors that might explain the discrepancies seen in our meta-analysis. The first criterion might involve the technique used – particularly with respect to the antibody used. While Rosenberg and Sui et al. used polyclonal p57^kip2^ antibodies at dilutions of 1:25 and 1:500 respectively, Khouja et al. used a monoclonal p57 ^kip2^ antibody at 1:500 dilutions. Whilst it is common knowledge that high specificity of monoclonal antibodies decreases cross-reactivity and background noise providing reproducible results and might explain the results by Khouja et al., it is unclear whether technique alone may explain the differences in the results of Rosenberg and Sui et al. Other variations in technique depending on the timing, type of autostaining technology employed (e.g. Dako/Ventana) may also account for the differences in results. This may also explain the differences in the p57^kip2^ in normal ovarian tissue which was observed by Sui & Guo et al. and none of the 10 cases in Khouja et al. series showed the expression. Only Sui & Guo et al. have assessed for expression in normal ovarian tissue and along the spectrum of ovarian tumours ranging from benign to borderline to malignant. While this gives an indication of p57^kip2^ expression across the tumour gradient, meaningful data analysis remains difficult from such small numbers.

Rosenberg et al. included only stage 2 & 3 EOC cases in their study. Since EOC tends to present as an advanced stage disease (>Stage 3) in more than 80% of cases excluding cases of stage 4 disease which represents a big cohort of the cases is unlikely to shed meaningful results especially when extensive drug development research is focused on advanced stage EOC in an attempt to improve survival. Due to more aggressive treatment (surgery & chemotherapy) survival in Stage 4 disease has definitely improved from the 13% 2 year survival mentioned in Rosenberg et al. to around 30-40% 5 year survival.

On the contrary, involving the early stages may shed light on the tumour development and biology which might improve our understanding of this silent killer disease and possibly assist in moving a step closer towards the ultimate dream of an ovarian cancer vaccine.

All 4 studies have clarified the grade of tumours as shown in Table 4. 3 out of the 4 studies focused on the adenocarcinomas and clearly describe the number of cases in each histological subtype though one wonders whether focusing on one subtype or histological sub-type might yield clarity of data especially in studies with small number of cases.

### Future work

The above studies interestingly have shown great variation in IHC expression of p57^kip2^ in EOC. This has obviously then led to differing conclusions being drawn as to the usefulness of this cell cycle regulator as an IHC marker for EOC. Conflicting results from the above studies may be explained by differences in technique, type of antibodies, case selection and different methodologies as described above. However, while the above studies incorporated the different histological subtypes of EOC, work on individual histological subtypes might provide more data. Multicentre collaborative studies might help to increase the number of cases especially for rarer tumours (e.g. clear cell). Combined IHC coupled with serum and RNA work to assess the full range of p57^kip2^ expressed in a wide variety of ovarian cancers along with a complete clinical dataset might help clarify the discrepancy from the above studies.

### p57^kip2^ as potential prognostic and therapeutic marker

Originally discovered as a CDKI, and considered as having only tumour suppressor function, it is becoming obvious that the role of p57^kip2^ has expanded beyond cell regulation. The above studies show that low p57^kip2^ function is associated with high grade, advanced clinical stage and overall poor prognosis [23]. This association may become crucial in target oriented cancer therapy by modulating p57^kip2^ levels. Several clinical trials including some Phase I & II clinical trials in haematological malignancies and some solid tumours are currently looking at reactivation of p57^kip2^ in cancer cells as a therapeutic strategy. This is based on the principle that downregulation of p57^kip2^ in the majority of cancers makes it an obvious tumour suppressor candidate [24]. As shown in various studies, suppression of the protein expression is due to the loss of p57^kip2^activity in cancer. Reactivation of p57^kip2^ expression can be a useful mechanism and some therapies (HDAC inhibitors and demethylating agents) that cause reactivation are already being used successfully.

## Conclusion

The involvement of p57^kip2^ is certainly more than just inhibition of cell growth as was previously thought. It is now known that its function includes promotion of cell differentiation and apoptosis and metastasis inhibition. In trying to unravel the mystery of p57^kip2^, its role as a diagnostic marker in the differential diagnosis of early hydatidiform mole has emerged. The four studies discussed above have suggested a role for p57^kip2^ as a prognostic marker at various levels and needs further clarification. With aggressive surgery being advocated for EOC including secondary debulking surgery, serial IHC work in conjunction with cell line and serum work from women at varying stages of treatment for EOC (both chemo-naïve and post treatment) may help to elucidate its role better. It may also help clarify its role in the development of drug resistance in women following first line platinum chemotherapy, both to assess potential response to treatment and to further explore if upregulation helps prevent development of resistance. Larger number of cases from each individual histological subtype needs to be investigated for meaningful data to emerge which might require collaborations. As a protein that appears to regulate various functions in the “cancer hallmarks cycle” resolving the enigma of its function might provide interesting results in terms of prognosis and therapy for EOC.

## Competing interest

All authors declare that they have no conflict of interest.

## Authors’ contributions

TKM and BH performed the literature search and extraction of papers. AJT, SBM and HC offered advice on relevant discussions. TKM and BH wrote the review and all authors have read and approved the final manuscript.
